# Supplementary material for: Analysis of human total antibody repertoires in TIF1γ autoantibody positive dermatomyositis
Source: Commun Biol. 2021 Mar 26;4:419. doi: 10.1038/s42003-021-01932-6 (PMC7997983; doi:10.1038/s42003-021-01932-6)
Supplement: Supplementary file 2 — Supplementary Information [file 42003_2021_1932_MOESM2_ESM.pdf]

Title

Analysis of human total antibody repertoires in TIF1γ autoantibody positive dermatomyositis

Spyridon Megremis[1^], Thomas D. J. Walker[2^], Xiaotong He[2], James O'Sullivan[1], William E.R. Ollier[3,4], Hector Chinoy[5,6], Neil Pendleton[7], Antony Payton[8], Lynne Hampson[2], Ian Hampson[2#], Janine A. Lamb[3,#*].

[1]Division of Evolution and Genomic Sciences, University of Manchester, Manchester, UK

[2]Division of Cancer Sciences, University of Manchester, Manchester, UK

[3]Division of Population Health, Health Services Research & Primary Care, University of Manchester, Manchester, UK

[4]Centre for Bioscience, Faculty of Science and Engineering, Manchester Metropolitan University, Manchester, UK

[5]National Institute for Health Research Manchester Biomedical Research Centre, Manchester University NHS Foundation Trust, University of Manchester, Manchester, UK

[6]Department of Rheumatology, Salford Royal NHS Foundation Trust, Manchester Academic Health Science Centre, Salford, UK

[7]Division of Neuroscience & Experimental Psychology, University of Manchester, UK

[8]Division of Informatics, Imaging & Data Sciences, University of Manchester, UK

^These authors contributed equally
[#]These authors jointly supervised this work

*Corresponding author: Janine.Lamb@manchester.ac.uk

Supplementary information

Supplementary information contains Supplementary methods, Supplementary figures 1-7, Supplementary tables 1-3, and Supplementary data files 1-3.

Supplementary Methods

Serum Epitope Repertoire Analysis (SARA) of DM patients

DM total Ig purification (M1); Competitive biopanning (M2); NGS epitope sequencing (M3). We retrieved ≈24 million and ≈36 million paired end FASTQ reads for HC individuals and DM patients respectively (Supplementary Figure 1a). These were highly enriched by associated NGS read counts and represent polyvalent FliTrx^{TM} peptide 12 AA coding sequences from DM and HC biopanned total Ig pools.

DM NGS data processing (M4); DM epitope signature set analysis (M5)

We retrieved 13,385,371 distinct expressed AA epitopes that represent the epitope signatures present within the DM and HC Ig pools (Supplementary Figure 1a). Associated NGS read frequencies reflected the epitope enrichment process achieved during biopanning, and Supplementary Figure 1b & 1c confirms that the 10-σ-99 NGS filter optimally controlled biopanning-induced sequencing noise to successfully provide 15,522 DM-associated and 4,817 HC-associated unique epitope sequences. Preferential enrichment for epitopes of length 11 and 12 AAs (Supplementary Figure 1d) confirmed genuine biopanning capture and is concordant with published peptide epitope ranges of 4 to 12 AAs (Buus et al., 2012; Hopp and Woods, 1981b). Minimum epitope lengths of 9AA applied with the 10-σ-99 NGS filter (above) proved optimal for annotation (Supplementary Figure 1e) using the unique sequence epitope sets of 15,522 for DM and 4,817 for HC individuals (Supplementary Figure 1f). Ig repertoire sequence overlap determined that only 0.65% of DM epitope set were present in healthy controls (fold-

change (FC) values of >=5x to 546x abundance vs HC individuals) while 6.44% of healthy epitope set was retained in the DM group (FC values of >=5.3x to 1240x vs DM) (Supplementary Figure 1f).

DM epitope annotation (M6) with phylogenetic and taxonomic interface (M7)

Our modified BLASTp approach retrieved 6.75 million (DM) and 2.25 million (HC) microbial or human protein annotations respectively which mapped to ≈14,000 microbial organisms. Enrichment plots revealed 4,994 *Distinct* infectious agents in HC individuals and 9,111 infectious agents in DM patients (Supplementary Figure 1g) of which 2,085 and 6,202 *Unique* infectious agents were identified in healthy individual and DM patients respectively (Supplementary Figure 1h). These signatures presented 377 highly enriched infectious agents in DM and 270 highly enriched agents in healthy individuals ranked by phylogeny (Supplementary Figure 1i).

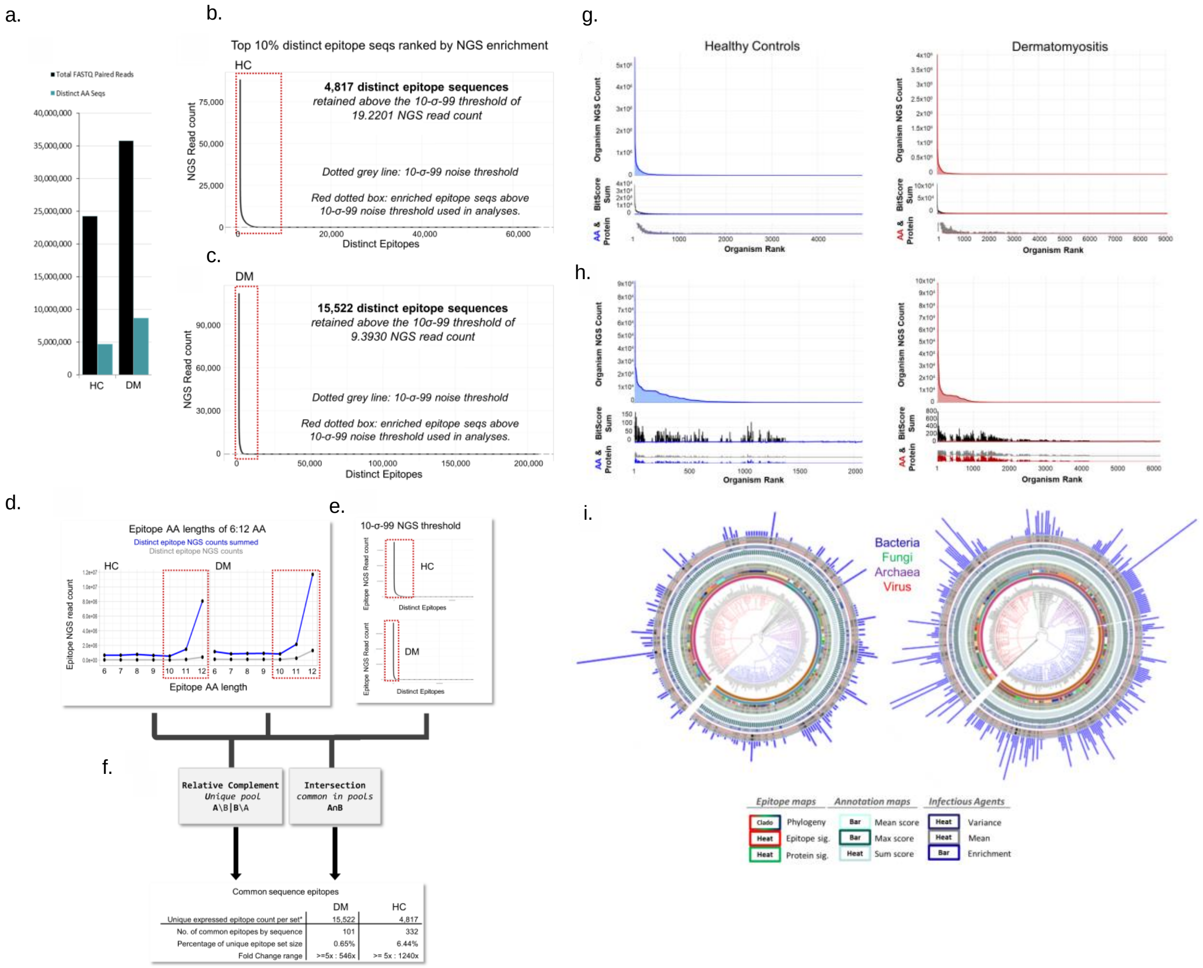

Supplementary figure 1: Immunoglobulin epitope enrichment in DM and HC.

NGS data processing: (a) Total NGS reads and expressed AA sequences per healthy control (HC) and dermatomyositis (DM) samples. Distinct epitope sequences ranked by NGS read count in (b) HC, and (c) DM patients; graphs are top 10% ranked epitopes per pools. The 10-σ-99 noise-floor is 10 standard deviations of the lowest 99% ranked epitope read counts per pool and prevents non-specific residual biopanning peptide sequences from entering future SARA modules. Red dotted boxes are proportions of clean epitope AA seq data for annotation. DM patients produced >three-fold enriched distinct epitope sequences vs HC (15,522 DM epitopes vs 4,817 HC). Epitope signature set analysis: (d) Epitope enrichment in HC and DM patients stratified by AA seq length. Blue: Summed NGS read counts per distinct AA epitope

sequence. Grey: Number of distinct AA epitopes. Preferential enrichment for epitopes lengths 10:12AA is clearly visible indicative of genuine antibody-epitope binding (biopanning). The DM pool preferentially enriched a larger cohort of disease-related epitope sequences c.f. HC. (e) 10-σ-99 NGS noise floor partitioning (red boxes are seqs carried downstream). (f) Relative complement and intersection sets. Only 101 (DM) and 332 (HC) distinct epitopes remained common to both pools after fold change control. Enrichment fold-change differences of common sequences were 5x:1,240x compared to opposing set (0.65% & 6.44% of respective unique epitope sets). (g) Enriched Distinct and Unique microbial agents in DM: Distinct and unique organisms. DM and HC microbes were annotated from 6,745,487 and 2,250,772 protein matches. (g) Images of interactive enrichment plots of highest ranked 4,994 distinct infectious agents (HC; left) and 9,111 infectious agents (DM; right). Top plot: NGS enrichment score per agent; middle plot: annotation confidence score; lower graphs: traces for epitope number and protein number per agent respectively. (h) Enrichment plots for 2,085 (HC) and 6,202 (DM) Unique infectious agents. (i) Images of interactive triage plots (legend provided). 377 highly enriched infectious agents mapped to DM and 270 highly enriched agents mapped to HC from ≈2.25 million & ≈ 6.75 million distinct epitopes. Inner two rings: trace data heat strips for epitope and protein number per agent respectively. Subsequent 3 rings: annotation confidence scores. Outermost three rings: 'per-infectious-agent' NGS enrichments of: mean NGS counts, NGS variance, final enrichment score (outermost bars).

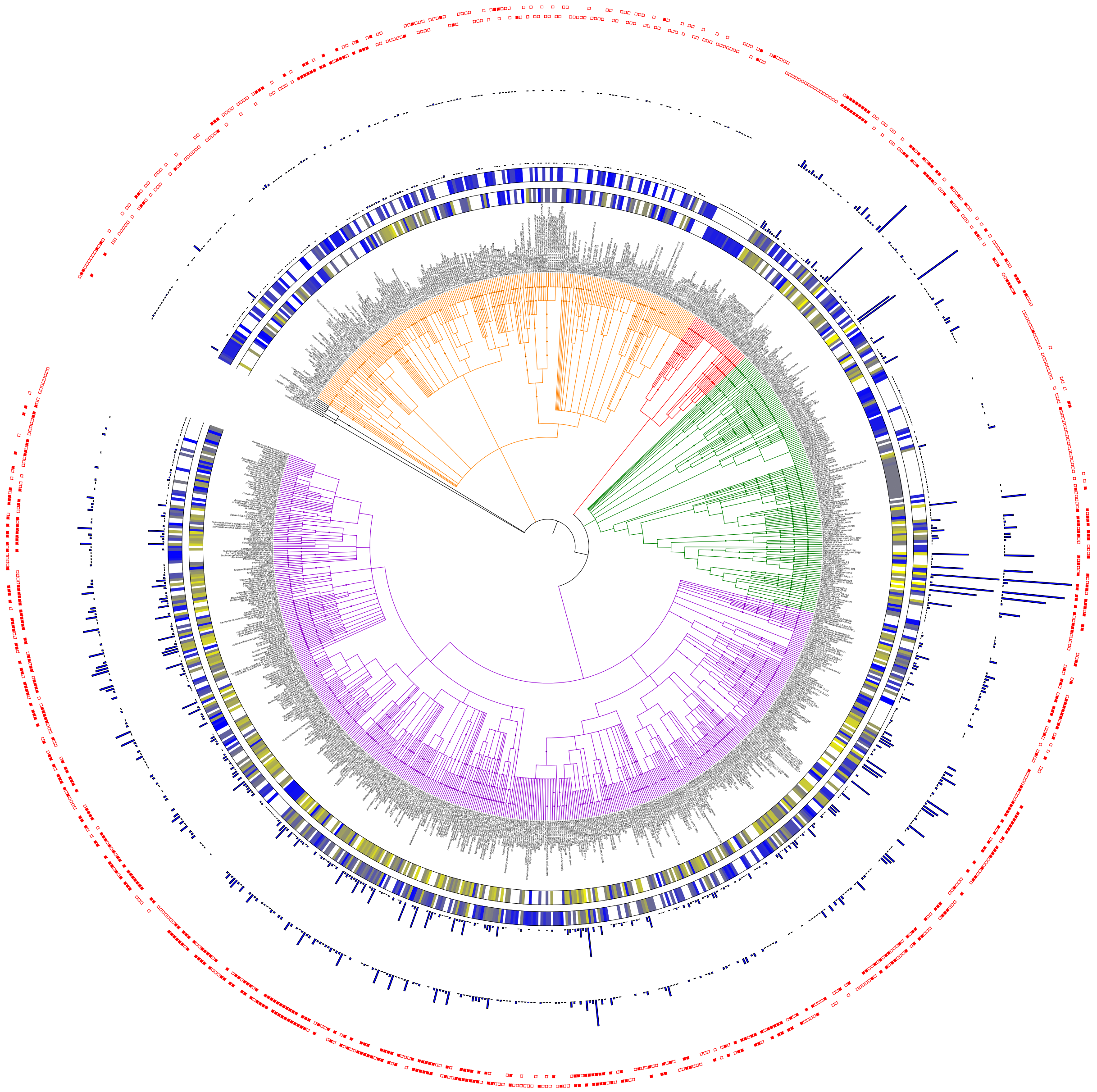

Supplementary Figure 2a

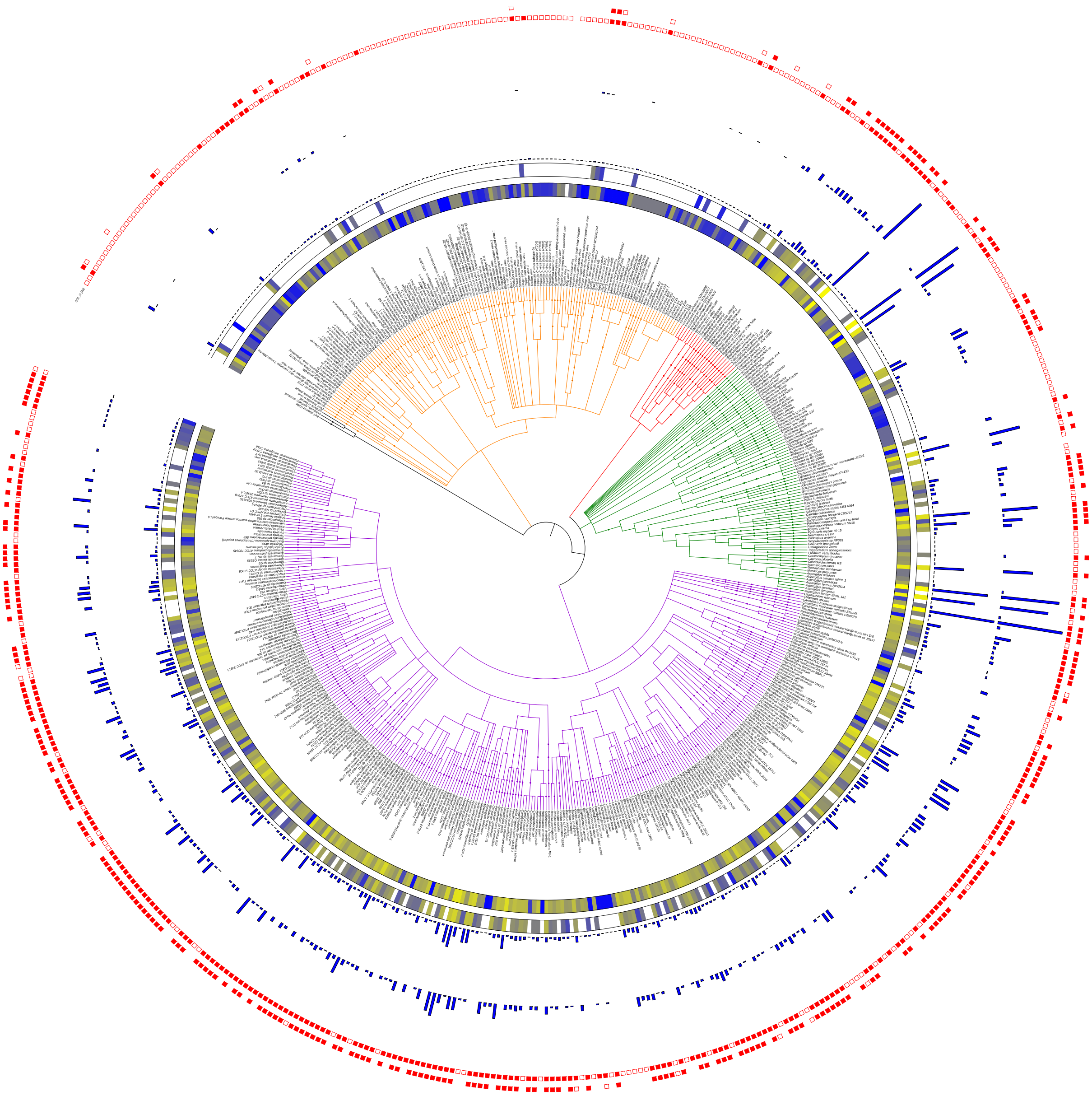

Supplementary Figure 2b

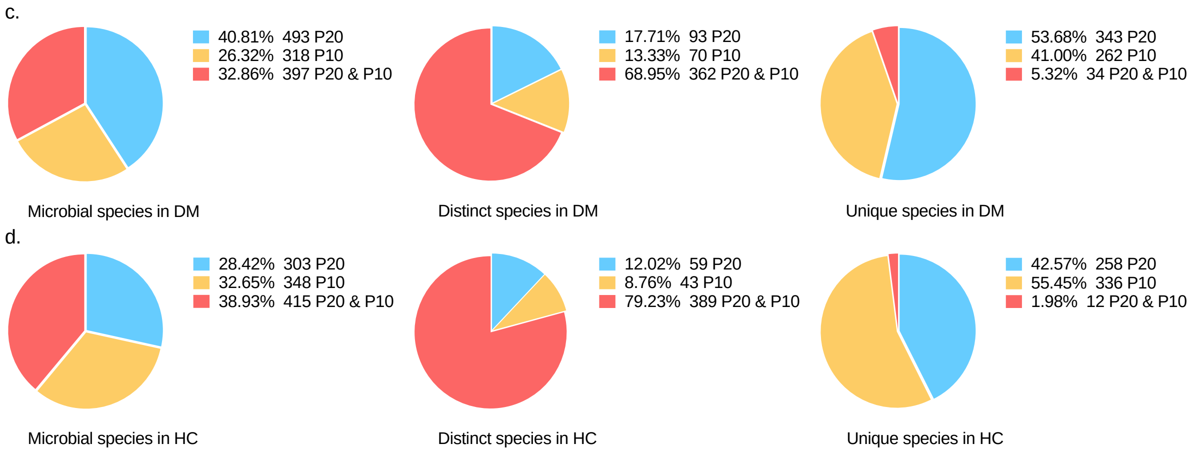

Supplementary Figure 2c & 2d

Supplementary figure 2: Antibodies against microbial species identified in DM and HC. Taxonomic clustering of identified microbial lineages up to the species level in (a) DM and (b) HC. The microbial cladograms contain taxa after integration of P10 and P20 experiments. Inner ring: Microbial NGS reads per taxon identified in P20. Second ring: Microbial NGS reads per taxon identified in P10; Colour-gradient: Yellow; highest amount, Blue; lowest amount. Microbial NGS reads are log10 transformed. Bar plots represent number of different AA peptide sequences per microbial taxon. Inner ring; P20, Outer ring: P10. Squares depict whether a microbial taxon was detected in the paired DM and/or HC: No filling; unique species. Red filling; distinct species. Squares annotated in both P20 and P10 represent a stably enriched microbial component in DM or HC. Inner ring; P20, outer ring: P10. Dendogram colours: Pink: bacteria, Orange: viruses, Green: eukaryotes, Red: Archaea. Distribution of shared microbial species between the P20 and P10 pools in DM and HC: In the pie chart panel the distribution of microbial species is presented in DM (c) and HC (d) pools. In dermatomyositis 32.86% of the total, 68.95% of distinct and 5.32% of unique species was shared between DM P20 and DM P10. 53.68% and 41% of unique species was observed only in DM P20 or DM P10 respectively. In the healthy group, 38.93% of total, 79.23% of distinct and 1.98% of unique species was shared between HC P20 and HC P10. 42.57% of unique species was identified only in HC P20 compared to 55.45% in HC P10.

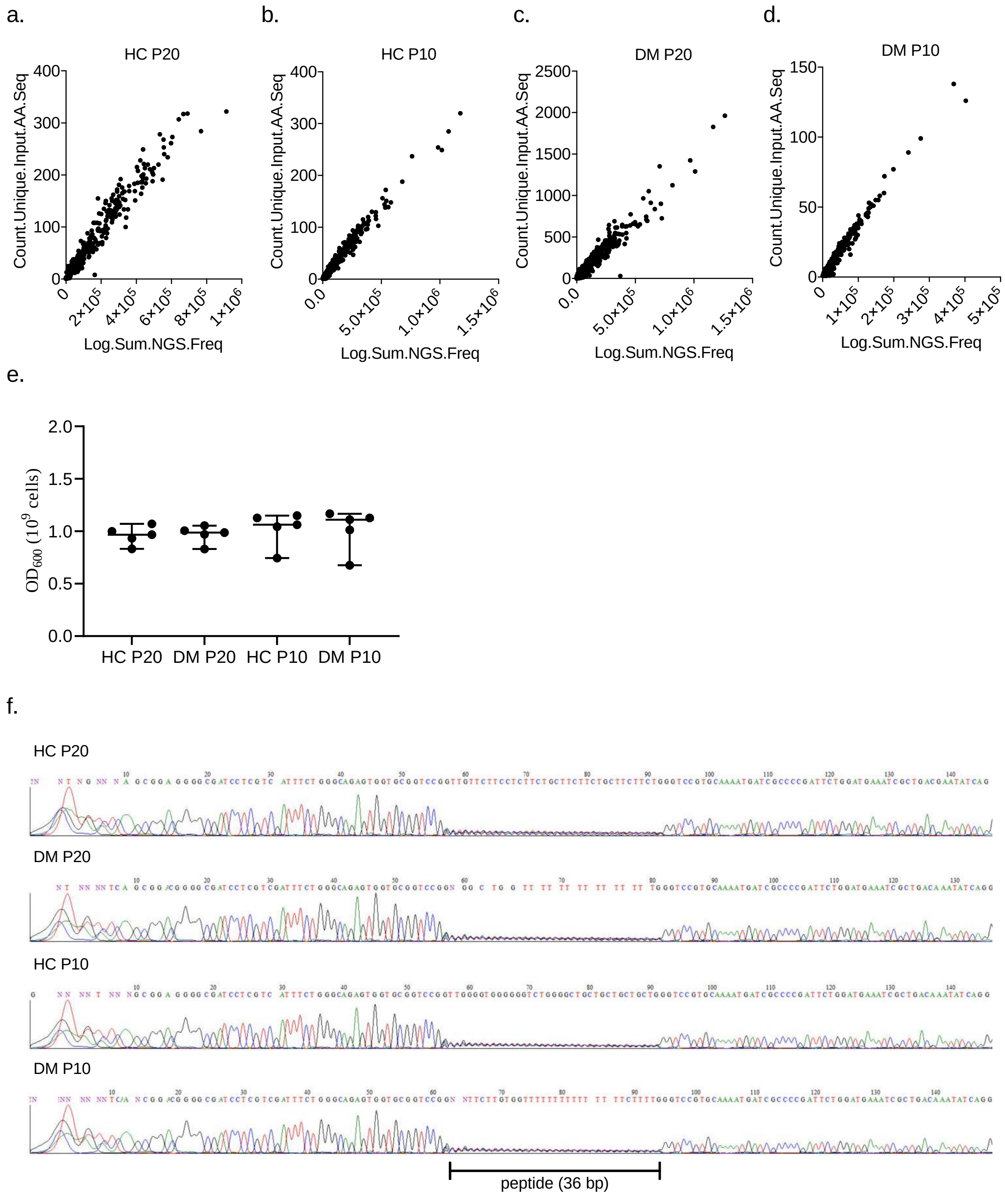

Supplementary figure 3: NGS data normalisation and verification of unbiased peptide display. Correlation of number of NGS reads and total number of epitopes per microbial species for (a) HC P20, (b) HC P10, (c) DM P20, and (d) DM P10.The scatter plots describe the positive and linear correlation of the sequencing depth and the number of identified microbial epitopes. To integrate both factors in the meta-analysis we defined $NGSR_{e\text{-}norm}$. (e) Optical density

measurements during the biopanning process. The optical density of the FliTrx E. Coli cells was measured after each round of panning (n=5). No differences were observed between the DM and HC cross-panning pairs. Welch's t test p value 0.861 for P20 comparison and 0.954 for P10. The median with 95%CI is annotated. (f) DNA chromatograph of amplified variance region. Illustrative variance region following Sanger sequencing. Portion of variance region is ¼ the intensities of flanking consensus along the 36bp variance region as a function of each measured base ("N") comprising equal quantities of A, C, T, and G nucleotides.

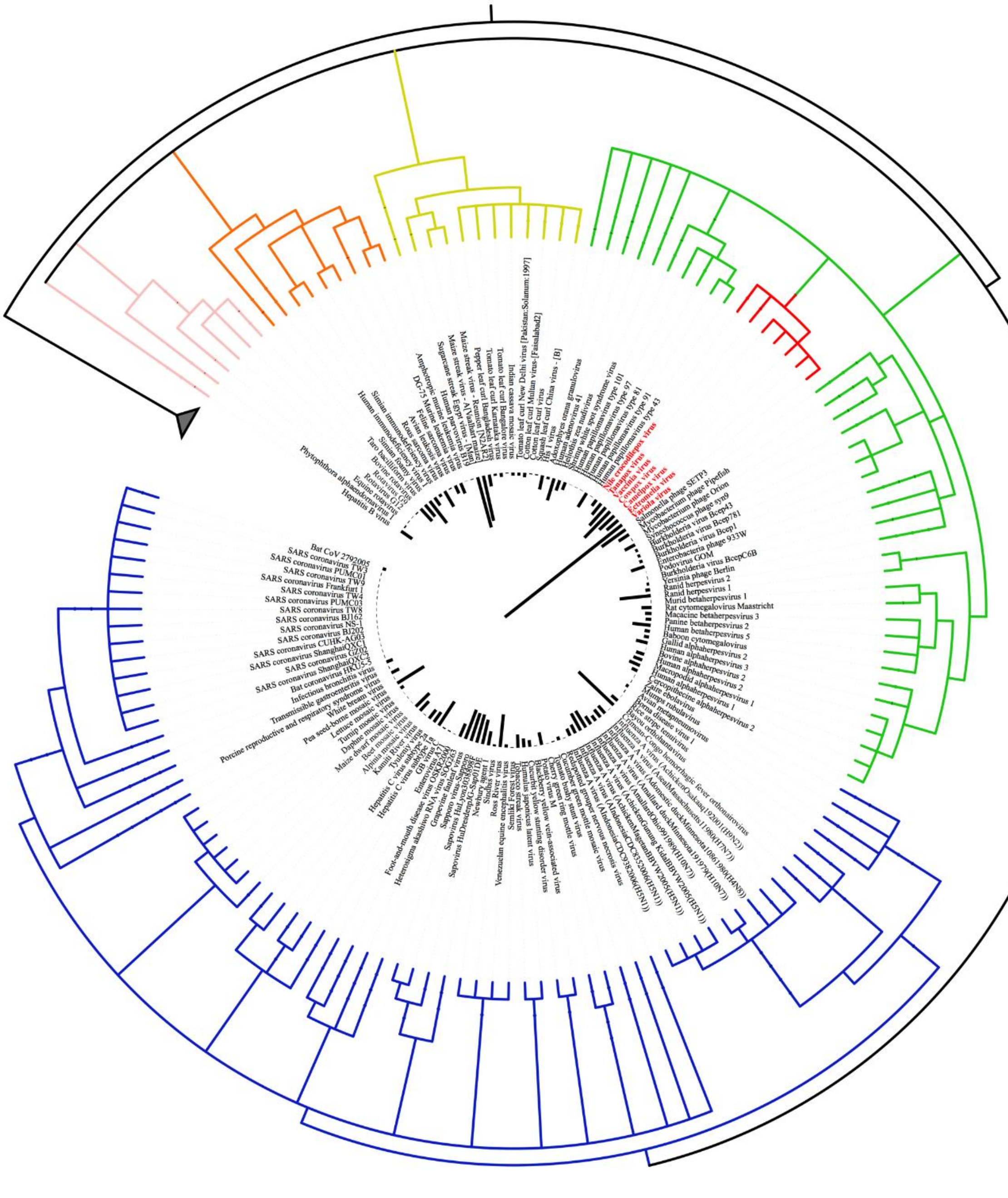

Supplementary figure 4: Dermatomyositis-specific antibodies targeting viral species. Inverted circular cladogram based on the taxonomic similarity of the dermatomyositis enriched viral species in DM P20. Clade colours are representative of genome type: Blue; single stranded RNA, Green; double stranded DNA, Gold; single stranded DNA, Orange; RNA reverse transcribing, Pink; DNA reverse transcribing. Nodes with red colour depict Chordopoxvirinae (Poxviridae) species. Single bar plots (inner ring) indicate $NGSR_{e\text{-}norm}$ values per viral species.

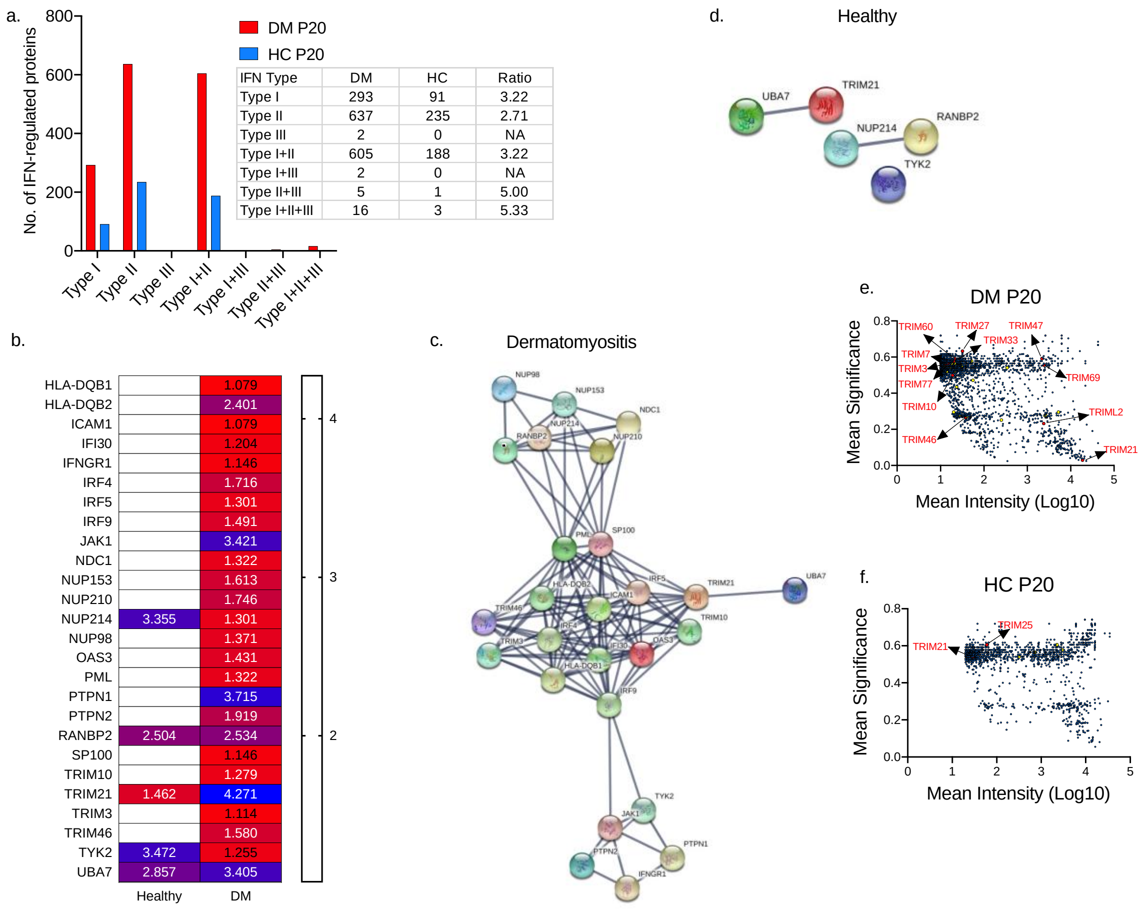

Supplementary figure 5: The IFN-related autoantibody proteome, focussing on IFNG. (a) Distribution of autoantibody protein-targets identified in DM and HC based on their predicted interferon-regulated profile. The majority of autoantibodies in DM target IFN-regulated proteins participating in type II alone or type I & II signalling pathways. (b) Heatmap of the autoantibody levels (mean log10 intensities) against proteins that are part of the IFNG signalling pathway in DM and HC. (c) Protein-protein interaction graph of autoantibody targets enriched in DM, and (d) enriched in the healthy sample. Each node represents an identified protein. Edges between two nodes represent confidence of interaction. Active interaction resources included experiments, gene fusion, co-occurrence, and co-expression. The minimum required score for a valid interaction was set to 0.700 (high confidence) (STRING 11.0)[1]. Disconnected nodes were hidden in the networks. Distribution of human protein antibody-targets (Blue nodes) based on the mean intensity (number of NGS reads) and mean significance

(BLASTp specificity) in (e) DM and (f) HC: Red colour; TRIM proteins, Yellow colour; IFNG-related proteins

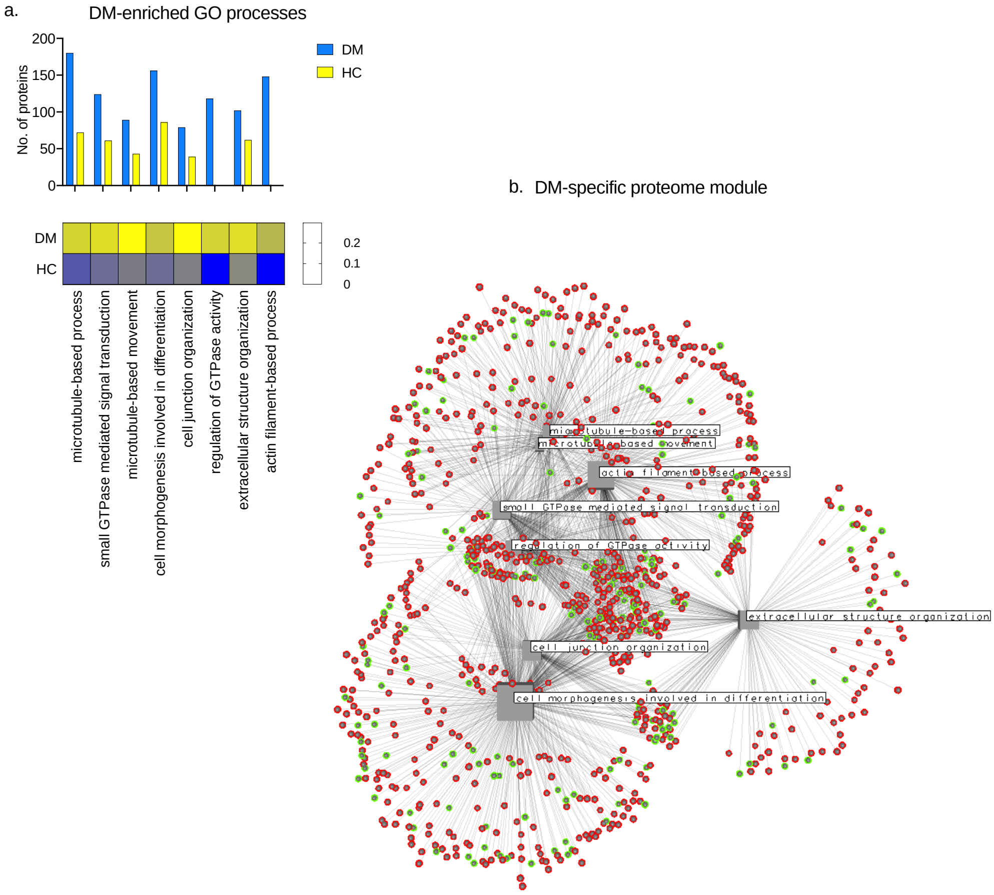

Supplementary figure 6: The dermatomyositis-associated autoantibody module. Human proteome autoantibody-targets identified in the DM P20 were used to screen for GO enrichment (biological processes). The distribution of proteins in GO processes in the DM-enriched autoantibody proteome and GO coverage (heatmap) in DM and HC is shown in (a). Graph representation of the DM proteome module (b): The GO processes are depicted as hubs (square nodes). Each hub is directly connected, through an edge, with the protein that is part of the specific GO process (circular nodes). Square node size is analogous to the number of interactions (edges). Nodes highlighted with red were exclusively identified in DM P20 (80% of nodes). The network contains 937 nodes (plus 8 GO hubs) with 1761 interactions. It is organised in one single component (module) with an average degree of interaction of 3.72. The eight biological functions were inter-linked because some of their protein members are shared.

The network was constructed and analysed using NAViGaTOR (Network Analysis, Visualization, & Graphing TORonto)[1].

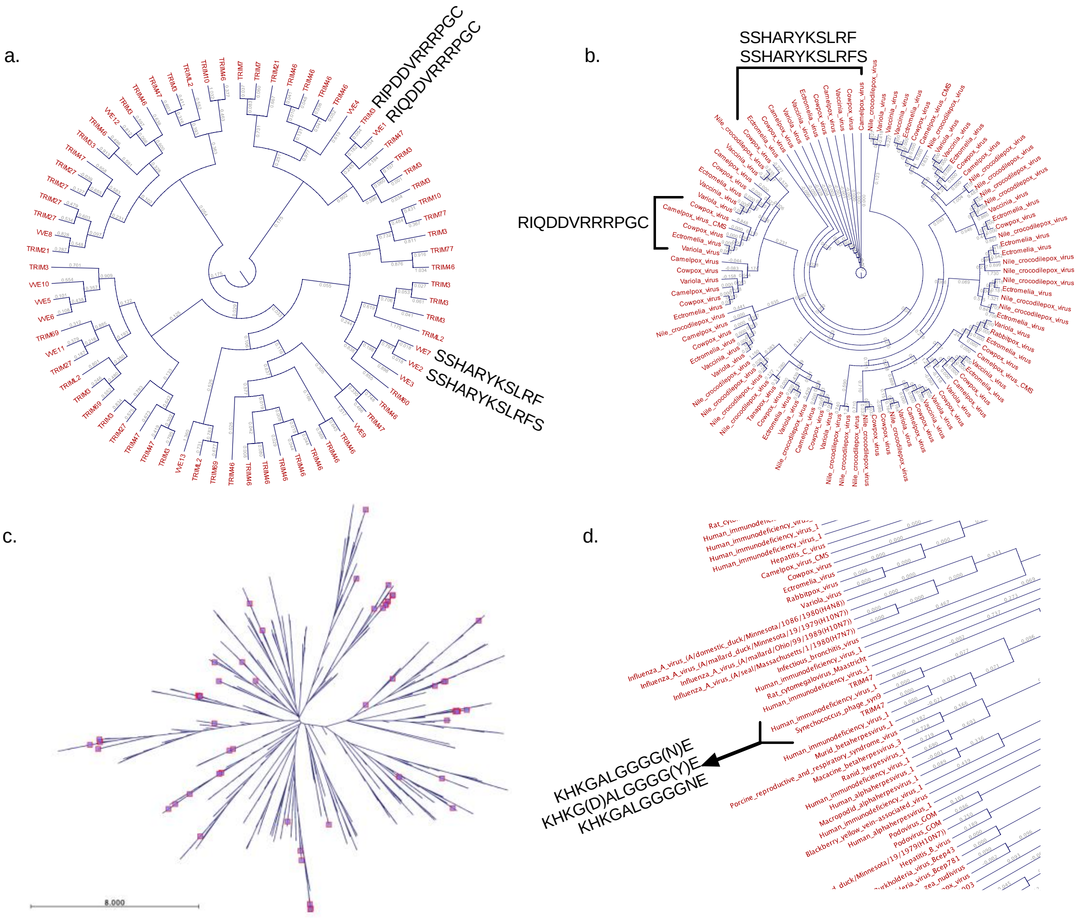

Supplementary figure 7: Cladograms of epitope sequence homology identified in DM. (a) Circular cladogram of *variola* virus and TRIM epitopes (high specificity thresholds). VVE; *variola* virus epitope. (b) Circular cladogram of Poxviridae epitopes identified in DM. The three motifs which were identified to be of high similarity between *variola* virus and TRIM3 are also shared across the Poxviridae family. (c) Radial cladogram of aligned viral and TRIM epitopes. The TRIM epitopes are annotated with squares. Interestingly, TRIM epitopes do not form clusters and they are spread across the tree. (d) Partial circular cladogram and phylogenetic distances amongst all viral and TRIM epitopes identified in DM. The *Human immunodeficiency virus 1*, *Synechococcus phage syn9* and TRIM47 share a common epitope (branch length=0.000). Branch lengths represent phylogenetic distance (Kimura protein distance).

ID	Gender	Diagnosis	Birth year	Age*	Race	Malignancy	Cancer associated myositis	Group
673	Male	DM	1963	47(44)	Caucasian	Negative		DM P10 & DM P20
318	Male	DM	1935	67(66)	Caucasian	Myeloma	Y	DM P10 & DM P20
630	Female	DM	1961	50(46)	Caucasian	Negative		DM P10 & DM P20
710	Female	DM	1957	54(42)	Caucasian	Negative		DM P10 & DM P20
196	Female	DM	1945	55(52)	Caucasian	Negative		DM P10 & DM P20
160	Female	DM	1941	59(55)	Caucasian	Breast	Y	DM P10 & DM P20
384	Female	DM	1946	60(54)	Caucasian	Negative		DM P10 & DM P20
215	Female	DM	1938	63(62)	Caucasian	Ovarian	Y	DM P10 & DM P20
419	Female	DM	1942	65(64)	Caucasian	Ovarian	Y	DM P10 & DM P20
695	Female	DM	1940	71(63)	Caucasian	Breast	Y	DM P10 & DM P20
679	Male	DM	1945	66(65)	Caucasian	Hepatic	Y	DM P20
641	Male	DM	1936	74(74)	Non-Caucasian	Lung	Y	DM P20
916	Female	DM/SSc	1958	53(49)	Caucasian	Negative		DM P20
146	Female	DM	1944	55(52)	Caucasian	Negative		DM P20
422	Female	DM	1953	55(55)	Caucasian	Negative		DM P20
432	Female	DM	1949	59(43)	Caucasian	Negative		DM P20
350	Female	DM	1943	61(61)	Caucasian	Ovarian	Y	DM P20
759	Female	DM	1948	63(35)	Caucasian	Negative		DM P20
446	Female	DM	1941	66(65)	Caucasian	Ovarian	Y	DM P20
621	Female	DM/SSc	1937	73(67)	Caucasian	Negative		DM P20
12637	Male	Control	1939	63	Caucasian	N/A		HC P10 & HC P20
12559	Male	Control	1938	64	Caucasian	N/A		HC P10 & HC P20
12796	Female	Control	1939	63	Caucasian	N/A		HC P10 & HC P20
12571	Female	Control	1937	65	Caucasian	N/A		HC P10 & HC P20

11247	Female	Control	1935	66	Caucasian	N/A		HC P10 & HC P20
10070	Female	Control	1933	68	Caucasian	N/A		HC P10 & HC P20
12206	Female	Control	1936	66	Caucasian	N/A		HC P10 & HC P20
12746	Female	Control	1936	66	Caucasian	N/A		HC P10 & HC P20
12328	Female	Control	1934	67	Caucasian	N/A		HC P10 & HC P20
12801	Female	Control	1934	67	Caucasian	N/A		HC P10 & HC P20
12478	Male	Control	1937	64	Caucasian	N/A		HC P20
11855	Male	Control	1937	65	Caucasian	N/A		HC P20
12409	Female	Control	1936	65	Caucasian	N/A		HC P20
12786	Female	Control	1937	65	Caucasian	N/A		HC P20
11680	Female	Control	1935	66	Caucasian	N/A		HC P20
12067	Female	Control	1936	66	Caucasian	N/A		HC P20
12627	Female	Control	1936	66	Caucasian	N/A		HC P20
10886	Female	Control	1935	67	Caucasian	N/A		HC P20
12415	Female	Control	1935	67	Caucasian	N/A		HC P20
12819	Female	Control	1934	67	Caucasian	N/A		HC P20

Supplementary Table 1: Clinical and demographic data for samples used in this study. DM; Dermatomyositis. SSc; systemic sclerosis. HC; healthy control. P10; pool of 10 samples. P20; pool of 20 samples. Cancer-associated myositis defined as cancer diagnosed within three years (before or after) of myositis onset. N/A; not available. *Age at time of sampling and age at DM onset.

Viral families	DM	HC
Coronaviridae	4.123	1.648
Geminiviridae	2.778	-0.474
Herpesviridae	2.105	0.738
Orthomyxoviridae	1.209	1.648
Poxviridae	0.984	-0.171
Potyviridae	0.760	0.132
Flaviviridae	0.536	1.648
Papillomaviridae	0.312	-0.171
Reoviridae	0.312	0.738
Caliciviridae	0.087	2.254
Podoviridae	0.087	-0.777
Prokaryotic	0.087	-0.474
Retroviridae	0.087	1.345
Togaviridae	0.087	
Myoviridae	-0.137	-0.171
Siphoviridae	-0.137	-0.171
Anelloviridae	-0.361	
Betaflexiviridae	-0.361	-0.474
Bromoviridae	-0.361	-0.474
Closteroviridae	-0.361	-0.474
Paramyxoviridae	-0.361	0.132
Picornaviridae	-0.361	3.466
Adenoviridae	-0.585	-0.777
Alloherpesviridae	-0.585	-0.171
Baculoviridae	-0.585	-0.171
Bornaviridae	-0.585	
Endornaviridae	-0.585	
Filoviridae	-0.585	-0.474
Hantaviridae	-0.585	
Marnaviridae	-0.585	
Nairoviridae	-0.585	
Nimaviridae	-0.585	
Nodaviridae	-0.585	
Nudiviridae	-0.585	
Parvoviridae	-0.585	0.132
Phenuiviridae	-0.585	
Pneumoviridae	-0.585	

Secoviridae	-0.585	-0.777
Tombusviridae	-0.585	-0.777
Unassigned	-0.585	
unclassified Arteriviridae	-0.585	
Alphaflexiviridae		0.435
Arenaviridae		-0.777
Arteriviridae		-0.777
Hepadnaviridae		-0.777
Iflaviridae		-0.777
Leviviridae		-0.777
Luteoviridae		-0.777
Microviridae		-0.777
Peribunyaviridae		-0.777
Polyomaviridae		-0.777
Tymoviridae		-0.474
Virgaviridae		0.132

Supplementary Table 2: Richness of antibodies targeting viral families identified in Dermatomyositis and healthy controls. The identified viral families are ordered based on decreasing richness (number of species per family) in DM and HC. Colour gradient is analogous to Z-transformed values of richness (Yellow: high, Blue: low). Missing values; Non-detected viral families.

Viral families	DM	HC
Nairoviridae	3.525	
Poxviridae	2.443	-0.215
Secoviridae	1.947	-0.285
Alloherpesviridae	1.966	-0.166
Caliciviridae	1.340	-0.137
Adenoviridae	1.203	-0.150
Geminiviridae	0.483	-0.080
Unassigned	0.420	
Marnaviridae	0.399	
Bromoviridae	0.126	-0.285
Nudiviridae	0.105	
Flaviviridae	0.068	-0.211
Orthomyxoviridae	0.052	-0.162
Prokaryotic	0.037	-0.191
Siphoviridae	0.033	-0.189
Potyviridae	0.000	0.040
Togaviridae	-0.013	-0.289
unclassified Arteriviridae	-0.100	-0.289
Retroviridae	-0.121	-0.227
Hantaviridae	-0.149	
Nimaviridae	-0.153	
Herpesviridae	-0.155	0.092
Betaflexiviridae	-0.295	-0.080
Podoviridae	-0.491	0.166
Reoviridae	-0.538	-0.163
Closteroviridae	-0.565	-0.279
Papillomaviridae	-0.569	-0.210
Myoviridae	-0.618	-0.179
Pneumoviridae	-0.621	
Baculoviridae	-0.659	-0.106
Endornaviridae	-0.762	
Tombusviridae	-0.797	-0.285
Bornaviridae	-0.808	
Coronaviridae	-0.808	-0.196
Parvoviridae	-0.814	-0.243
Picornaviridae	-0.822	-0.251
Paramyxoviridae	-0.850	-0.122
Nodaviridae	-0.854	

Filoviridae	-0.855	-0.286
Anelloviridae	-0.865	
Phenuiviridae	-0.869	
Alphaflexiviridae		-0.215
Arenaviridae		0.010
Arteriviridae		-0.053
Hepadnaviridae		-0.267
Iflaviridae		0.270
Leviviridae		-0.100
Luteoviridae		-0.287
Microviridae		-0.126
Peribunyaviridae		-0.286
Polyomaviridae		6.193
Tymoviridae		0.036
Virgaviridae		-0.200

Supplementary Table 3: Antibody mean NGSRe-norm of viral families identified in Dermatomyositis and healthy controls. The identified viral families are ordered based on decreasing antibody mean NGSRe-norm in DM. Colour gradient is analogous to log10 transformed values of mean NGSRe-norm (Yellow: high, Blue: low). Missing values; Non-detected viral families.

Reference

1. Brown, K. R. et al. NAViGaTOR: network analysis, visualization and graphing Toronto. Bioinformatics **25**, 3327–3329 (2009).
