# Supplementary material for: Analysis of human total antibody repertoires in TIF1γ autoantibody positive dermatomyositis
Source: Commun Biol. 2021 Mar 26;4:419. doi: 10.1038/s42003-021-01932-6 (PMC7997983; doi:10.1038/s42003-021-01932-6)
Supplement: Supplementary file 3 — Description of Additional Supplementary Files [file 42003_2021_1932_MOESM3_ESM.pdf]

Description of Additional Supplementary Files

File name: Supplementary Data 1

Description: Includes the underlying data presented in all figures and supplementary figures.

File name: Supplementary Data 2

Description: Microbial annotations and accompanying information.

File name: Supplementary Data 3

Description: Human protein annotations and accompanying information.
