# Supplementary material for: Analysis of human total antibody repertoires in TIF1γ autoantibody positive dermatomyositis
Source: Commun Biol. 2021 Mar 26;4:419. doi: 10.1038/s42003-021-01932-6 (PMC7997983; doi:10.1038/s42003-021-01932-6)
Supplement: Supplementary file 7 — Reporting Summary [file 42003_2021_1932_MOESM7_ESM.pdf]

nature research

Corresponding author(s): Janine Lamb. COMMSBIO-20-1129B

Last updated by author(s): 2021/02/01

Reporting Summary

Nature Research wishes to improve the reproducibility of the work that we publish. This form provides structure for consistency and transparency in reporting. For further information on Nature Research policies, see our Editorial Policies and the Editorial Policy Checklist.

Statistics

For all statistical analyses, confirm that the following items are present in the figure legend, table legend, main text, or Methods section.

n/a	Confirmed	
☐	☒	The exact sample size (n) for each experimental group/condition, given as a discrete number and unit of measurement
☒	☐	A statement on whether measurements were taken from distinct samples or whether the same sample was measured repeatedly
☐	☒	The statistical test(s) used AND whether they are one- or two-sided<br>*Only common tests should be described solely by name; describe more complex techniques in the Methods section.*
☒	☐	A description of all covariates tested
☐	☒	A description of any assumptions or corrections, such as tests of normality and adjustment for multiple comparisons
☐	☒	A full description of the statistical parameters including central tendency (e.g. means) or other basic estimates (e.g. regression coefficient) AND variation (e.g. standard deviation) or associated estimates of uncertainty (e.g. confidence intervals)
☐	☒	For null hypothesis testing, the test statistic (e.g. F, t, r) with confidence intervals, effect sizes, degrees of freedom and P value noted<br>*Give P values as exact values whenever suitable.*
☒	☐	For Bayesian analysis, information on the choice of priors and Markov chain Monte Carlo settings
☒	☐	For hierarchical and complex designs, identification of the appropriate level for tests and full reporting of outcomes
☒	☐	Estimates of effect sizes (e.g. Cohen's d, Pearson's r), indicating how they were calculated

Our web collection on statistics for biologists contains articles on many of the points above.

Software and code

Policy information about availability of computer code

Data collection	FliTrx peptide surface display system. Ilumina NextSeq Sample preparation and sequencing. The custom Python code supporting the current study have not been deposited in a public repository but will be deposited following submission of our methods manuscript (in preparation). In the intervening period these will be available from the corresponding author on request.
Data analysis	Software and resources used: R 3.2-3.6; BLASTp; NCBI Taxonomy database; PhyloT biobyte; Interactive Tree of Life (iTOL); Metscape; KEGG; UniProt; The Human Protein Atlas; CLC Genomics Workbench 12; ViralZone root-ExPASy; Interferome v2.01; STRING 11.0; NAViGaTOR (Network Analysis, Visualization, & Graphing TORonto); GraphPad Prism; SuperPath; GeneCardsSuite: PathCards. The custom Python code supporting the current study have not been deposited in a public repository but will be deposited following submission of our methods manuscript (in preparation). In the intervening period these will be available from the corresponding author on request.

For manuscripts utilizing custom algorithms or software that are central to the research but not yet described in published literature, software must be made available to editors and reviewers. We strongly encourage code deposition in a community repository (e.g. GitHub). See the Nature Research guidelines for submitting code & software for further information.

Data

Policy information about availability of data

All manuscripts must include a data availability statement. This statement should provide the following information, where applicable:

- Accession codes, unique identifiers, or web links for publicly available datasets
- A list of figures that have associated raw data
- A description of any restrictions on data availability

Raw sequencing data have been deposited in DRYAD (https://doi.org/10.5061/dryad.gmsbcc2mb). Human proteins and microbial annotations can be found in DRYAD (https://doi.org/10.5061/dryad.gmsbcc2mb). Source data can be found in Supplementary Data files 1-3. All other data are available from the corresponding

author on reasonable request.

Field-specific reporting

Please select the one below that is the best fit for your research. If you are not sure, read the appropriate sections before making your selection.

☒ Life sciences ☐ Behavioural & social sciences ☐ Ecological, evolutionary & environmental sciences

For a reference copy of the document with all sections, see nature.com/documents/nr-reporting-summary-flat.pdf

Life sciences study design

All studies must disclose on these points even when the disclosure is negative.

Sample size	Preliminary analyses of data obtained from pooled sera from 10 women with early stage ovarian cancer compared to 10 healthy controls identified sequences from a number of organisms found exclusively or more commonly in women with ovarian cancer. Validation was carried out using a synthetic peptide array in an independent sample, confirming that a pooled sample size of 10 was sufficient.
Data exclusions	No data were excluded from analyses.
Replication	These findings have not been replicated. Preliminary analyses in a separate ovarian cancer cohort demonstrated that the results were replicable using a synthetic peptide array in an independent sample.
Randomization	Sample randomization was not carried out in this study. Samples from individuals with dermatomyositis were compared to age and gender matched healthy controls.
Blinding	Blinding was not relevant to this study as competitive bio-panning was applied to total immunoglobulin fractions from pooled plasma of dermatomyositis patients vs healthy controls.

Reporting for specific materials, systems and methods

We require information from authors about some types of materials, experimental systems and methods used in many studies. Here, indicate whether each material, system or method listed is relevant to your study. If you are not sure if a list item applies to your research, read the appropriate section before selecting a response.

Materials & experimental systems

n/a	Involved in the study	
☒	☐	Antibodies
☒	☐	Eukaryotic cell lines
☒	☐	Palaeontology and archaeology
☒	☐	Animals and other organisms
☐	☒	Human research participants
☒	☐	Clinical data
☒	☐	Dual use research of concern

Methods

n/a	Involved in the study	
☒	☐	ChIP-seq
☒	☐	Flow cytometry
☒	☐	MRI-based neuroimaging

Human research participants

Policy information about studies involving human research participants

Population characteristics	Plasma samples were collected from anti-TIF1 positive adult-onset dermatomyositis patients. All individuals fulfilled definite or probable Bohan and Peter classification criteria for dermatomyositis. Gender and age matched (at time of sample collection) healthy controls were identified through the University of Manchester Longitudinal Study of Cognition in Normal Healthy Old Age cohort.
Recruitment	Dermatomyositis patients were collected through the UK Myosiitis Network
Ethics oversight	All samples were collected with relevant research ethics committee approval (MREC 98//8/86 North West Haydock Research Ethics Committee for UKMyoNet and UREC 02225 and UREC4 2017-1256-2489 for healthy control cohort). Study participants provided written informed consent.

Note that full information on the approval of the study protocol must also be provided in the manuscript.
